# P. P. Johnson

**Published:** 1911-03

**Authors:** 


### P. P. JOHNSON, M.B., C.M.Edin., of Yatton.

We regret to record the death of Dr. P. P. Johnson, of Yatton, after a long and painful illness, borne with much fortitude. Dr. Johnson was 57 years of age, and was educated at Sedbergh, afterwards proceeding to Edinburgh University, where he took his degree in 1877. He practised for over nine years at Littleborough, in Lancashire, and for a short time in Starbotton, Yorkshire, and came to Yatton in 1889.

He was a thoroughly good all-round practitioner, and his close attention to his work and absolute forgetfulness of self in his care for the sick under his charge earned for him the confidence and affection of the district in which he practised. Striking evidence of the esteem that he enjoyed was afforded by the very large attendance at his funeral, and the general regret expressed for his loss.

He was a most genial man, and took an active part in all movements for promoting the social life of the village and its welfare; he was largely concerned in the provision of a good supply of water.

The Freemasons, Oddfellows, Church of England Men's Society, Yatton Cricket Club, Conservative Association, and Parish Council were all represented at his funeral, and indicate the wide extent of his interests outside his professional work.

He leaves a widow, one son, and four daughters to mourn his loss.

---

### Local Medical Notes.

